# A Novel Technique Identifies Valve-Like Pathways Entering and Exiting Schlemm’s Canal in *Macaca nemestrina* Primates With Similarities to Human Pathways

**DOI:** 10.3389/fcell.2022.868029

**Published:** 2022-07-04

**Authors:** Elizabeth A. Martin, Murray A. Johnstone

**Affiliations:** ^1^ Department of Ophthalmology, Indiana University School of Medicine, Indianapolis, IN, United States; ^2^ Department of Ophthalmology, University of Washington School of Medicine, Seattle, WA, United States

**Keywords:** cell adhesive mechanism, intraocular pressure (IOP), aqueous outflow and resistance, aqueous valves, glaucoma, trabecular meshwork (TM), pulsatile aqueous outflow, cell junctions

## Abstract

**Purpose:** The aim of the study was 1) to describe a novel combination of techniques that permit immunohistochemistry imaging of Schlemm’s canal inlet (SIV) and outlet (SOV) valve-like structures, 2) to identify tissue-level SIV adhesive relationships linking the trabecular meshwork (TM) to hinged collagen leaflets at the Schlemm’s canal (SC) external wall, and 3) to determine whether the SIV lumen wall’s adhesive vascular markers are similar to those of the SC inner wall endothelium.

**Materials and Methods:** Anterior segments of 16 *M. nemestrina* primates underwent immunohistochemistry (IHC) labeling. We perfused fluorescent microspheres into 12 of the eyes. Limbal tissues were divided into quadrants, viscoelastic introduced into SC, tissues fixed, immunohistochemistry performed, radial segments cut, tissues clarified, and confocal microscopy performed. Finally, we generated ImageJ 3D projections encompassing the TM, SC, and distal pathways.

**Results:** IHC imaging identified 3D relationships between SIV, collector channel ostia, collector channels (CC), SOV, and intrascleral channels. Imaging depth increased 176.9%, following clarification (*p* < 0.0001). Imaging demonstrated CD31, collagen type 1 and 4 in the walls of the SIV lumen and more distal pathways. In eight eyes, 384 segments were examined, 447 SIV identified, and 15.4% contained microspheres.

**Conclusion:** Our technique’s imaging depth permitted the identification of SIV linkage between the TM and SOV. We found comparable cell–cell adhesion molecules (CD31) and basement membrane components in the SC inner wall and SIV lumen walls. Recent OCT studies have suggested that SIV tensional relationships may control CC entrance dimensions that regulate distal resistance. Cellular adhesive properties sustain SIV tensional relationships. These SIV cell–cell and cell-basement membrane properties warrant further study because abnormalities could be a factor in the IOP elevation of glaucoma.

## Introduction

Glaucoma is the second leading cause of blindness, estimated to affect 60 million people worldwide (Quigley and Broman, 2006). Intraocular pressure (IOP) is the only modifiable risk factor for glaucoma (Broman et al., 2008; Coleman and Miglior, 2008). The balance of aqueous humor production and outflow determines IOP, and active regulation resides in the outflow side of the system. The conventional aqueous humor outflow pathway begins at the trabecular meshwork (TM) and then aqueous humor enters Schlemm’s canal (SC), followed by the entry into collector channels (CCs), leading to episcleral veins.

The TM contributes to outflow resistance, but it is not the only region controlling resistance in the outflow system. Our glaucoma patients demonstrate this point because if TM was the only source of resistance, a surgical goniotomy would be the definitive treatment for glaucoma (Grover et al., 2018). Unfortunately, SC-directed minimally invasive glaucoma surgery (MIGS) procedures sometimes fail, requiring a more complex assessment of resistance distal to the TM.

The TM attaches anteriorly to the Schwalbe’s line and posteriorly to the scleral spur and the ciliary body. The force of IOP causes the TM to move outward into SC, causing it to assume a distended configuration (Johnstone and Grant, 1973; Grierson and Lee, 1975). The IOP forces the TM outward into SC, but the counterbalancing force of ciliary body tension and elastance properties of the TM lamellae prevent collapse into SC. The balanced forces thus serve to optimally position the TM in three-dimensional (3D) space between the anterior chamber and the canal (Kaufman and Bárány, 1976; Rohen et al., 1981; Lütjen-Drecoll et al., 1988).

### Tissue and Cell–Cell Adhesive Relationships in the Outflow System

Studies in living primates exposed to physiologic pressures demonstrate that cytoplasmic processes link the TM lamellae, the juxtacanalicular region, and SC inner wall endothelium together (Johnstone and Grant, 1973; Grierson and Lee, 1974; Grierson and Lee, 1975; Grierson et al., 1978; Johnstone, 1979). Similarly, cellular elements are present distal to the TM. SC inlet valve-like structures (SIV) arise from the SC endothelium in a funnel-like configuration and then merge into a cylindrical tube-like region that attaches to the SC outer wall (Johnstone, 1974; Johnstone et al., 2021). The cylindrical tube-like structure and its dual attachment, from the internal wall to the external wall of SC, becomes a functional valve-like system with relevant properties documented in prior reports of humans and non-human primates (Johnstone, 1974; Johnstone, 2004; Hariri et al., 2014; Johnstone et al., 2022).

The SIV attachments link the SC inner wall and its attached trabecular lamellae to hinged flaps at CC entrances (Johnstone, 1974; Smit and Johnstone, 2002; Johnstone, 2004; Hariri et al., 2014; Xin et al., 2021). The hinged flaps adjacent to CC are a component of septa, which are oriented circumferentially along the SC external wall. The septa form the boundary between the SC lumen and a more distal circumferentially oriented deep vascular plexus (CDSP) adjacent to SC (Hariri et al., 2014; Johnstone et al., 2016; Xin et al., 2016; Xin et al., 2017).

The hinged CC flaps respond to pressure changes and act as SC outlet valve-like structures (SOV) (Hariri et al., 2014). The SIV provide connections between the TM and SOV, resulting in synchronous movement of the structures (Xin et al., 2017). When the TM responds to IOP changes, the adhesive attachments of the SIV at the tissue and cellular level can open and close the CC entrances (Hariri et al., 2014; Xin et al., 2016). The pressure-dependent tissue responses of these outflow pathways demonstrate the importance of exploring distal attachment mechanisms. MIGS procedures also require knowledge of distal resistance to optimize surgical decisions, making an improved understanding essential to optimal glaucoma patient care (Richter and Coleman, 2016).

### Extracellular and Cellular Adhesive Mechanisms

Cellular–extracellular adhesions are possible pressure sensors with a potential role in glaucoma, and integrin adhesive junctions are the subject of intensive study (Filla et al., 2019). During pressure-dependent extracellular matrix stretching, there is potential to disrupt binding between fibronectin and α5β1 integrin. The disruption can alter signaling and change the 3D architecture of the extracellular matrix (Faralli et al., 2019).

Cell–cell adhesion mechanisms are also highly relevant to resolving pressure-related problems in glaucoma (Webber et al., 2018). SCE tight junctions are well-characterized and contain abundant VE-cadherin and components of adherens junctions; occluding and gap junction formations require such junctional components (Wallez and Huber, 2008). The endothelial cell–cell attachment, PECAM (CD31), is another crucial multifunctional adhesive and signaling molecule (Newman and Newman, 2003), one involved in defining SCE as a vascular endothelium (Stamer et al., 1998). The endothelial walls of the lumen of the SIV spanning SC and their ability to control SOV depend on the maintenance of optimized cell–cell adhesive attachments, so establishing their composition is essential.

### Schlemm’s Canal Dilation and Clarification: Crucial Factors in Assessing Distal Outflow Mechanisms

Dilation of SC is essential for assessing adhesive relationships between the TM, SIV, and SOV. Canal dilation can be reliably carried out by three techniques: 1) reversal of pressure gradients in living primates followed by *in vivo* fixation (Johnstone and Grant, 1973), 2) viscoelastic injection followed by fixation (Smit and Johnstone, 2002), and 3) continuous infusion of BSS into SC in fresh tissue during OCT imaging (Hariri et al., 2014). Immunohistochemistry (IHC) used to characterize adhesive constituents involves confocal microscopy (CFM), a technique with a small field of view and a limited depth of field. Thin tissue sections are often necessary because light scattering markedly limits the ability of CFM to sample weakly emitting labels used in IHC (Elliott, 2020). However, SIV geometric relationships and cell–cell adhesive molecule assessments require regional 3D volumetric imaging.

Because limbal tissues are dense and opaque, light entering and passing through specimens becomes scattered, reflected, refracted, and absorbed. These factors reduce the intensity of laser light and decrease the optical quality as increased penetration into the tissue is attempted (Zucker et al., 1999). Investigators report several techniques for increasing tissue transparency to attain a greater imaging depth. Agents include FocusClear, Scale, glycerol (Hama et al., 2011), and benzyl alcohol–benzyl benzoate (BABB) (Gard, 1993). Studies have not systematically compared the practicality of using these agents in terms of rapidity of clarification, retention of the IHC label, transmission properties, tissue shrinkage, and cost.

### Study Goals

The purpose of this report was to describe tools that permit the study of relationships and constituent properties of outflow structures distal to the TM. Relationships dependent on the adhesive properties of the cellular components are of particular interest. Specific aims to study relationships include combining the use of four techniques: 1) SC dilation with viscoelastic, 2) clarification of the tissues for enhanced depth of confocal imaging, 3) use of the cellular adhesion and basement membrane molecule labels, CD31, collagen type 1 (Col Type 1), and collagen type 4 (Col Type 4) to assess structural relationships, composition, and connections of pathways distal to the TM, and 4) introduction of fluorescent tracers to assess the SIV lumen patency and ability to transfer the fluid from the juxtacanalicular region to SC and distal channels. Our goal was to determine whether the development of this combination of techniques will provide an approach for future more comprehensive studies of cell–cell and cell–ECM adhesive mechanisms in the tissues distal to the TM.

## Materials and Methods

Bilateral anterior segments of 16 *M. nemestrina* primate eyes (two for clarification studies, and 14 for microsphere perfusion) were obtained. At necropsy, while still *in vivo*, the 12 o’clock limbal position was marked with tissue dye to ensure identification of individual quadrant orientation. We obtained the anterior segments after a team did hemisection in the necropsy room. Immediately after enucleation, the conjunctiva was removed, the eyes were bisected in the anterior–posterior plane, and the vitreous was removed with cellulose sponges, leaving the crystalline lens intact.

### Perfusion

The anterior segment was mounted on an organ culture perfusion device, and a 30-g needle connected to PE 60 tubing was inserted into the anterior chamber (AC). The other end of the cannula was connected to a reservoir that provided initial control of IOP so that later insertion of a larger needle did not excessively deform the globe. The firm eye then permitted the insertion of two 23-gauge needles. A fitting in the perfusion device led to the posterior chamber, which was connected to another reservoir. The anterior and posterior chambers were initially set to equal pressures of 24 mmHg. We perfused fluorescent microspheres (FMS) into the anterior chamber by setting one reservoir 1 mm above and the other 1 mm below the 24 mm mean pressure.

The reservoir relationships permitted the maintenance of mean pressures approximating 24 mmHg while at the same time permitting microsphere perfusion into the chamber. We performed preliminary perfusions with various sizes of 0.02% concentration of carboxylate-modified microspheres (FluoSpheres™). Sphere sizes perfused were as follows: 15, 10, 8, 5, 2, 1µm, 500, and 200 nm. These preliminary studies demonstrated that 1 μm and 500 nm MS provided optimal filling of outflow system structures where FMS were found in the TM, SIV, and distal outflow structures. Final perfusions were with 1 µm (*n* = 5) or 500 nm MS (*n* = 3) for 25 min at a mean IOP of 24 mmHg.

### Preparation for Immunohistochemistry

Once the microsphere perfusion was complete, the anterior segment was removed from the perfusion apparatus, the lens was extracted, and the anterior segment was divided into four labeled quadrants. Each quadrant was trimmed to include a 3-mm long wedge centered on the limbus. The tips of 31 g needles pinned the quadrants onto the surface of a layer of paraffin in phosphate buffer-containing Petri dishes to provide stability for further dissection. Quadrant stability permitted controlled, gentle removal of the ciliary body and iris using forceps and Westcott scissors. Special care was taken to cut the ciliary body–TM connections to avoid stripping away the regions of the TM normally attached to the ciliary body.

### Viscoelastic Dilation of Schlemm’s Canal and Segment Preparation

PE 60 tubing was heated and pulled to a small diameter, creating infusion cannulas with an outside diameter of 150 µm. The cannulas were inspected to ensure an open end and a steep taper to provide occlusion of the canal at the insertion site. Next, viscoelastic was mixed with 1% toluidine blue solution to make a 0.1% toluidine blue viscoelastic mixture. Tinted viscoelastic (VE) was then loaded in a 3cc syringe, and the proximal end of the PE 60 tubing (diameter ID 0.76 mm × OD 1.22 mm) was attached to the syringe, with the distal end tapered to 0.150 mm OD.

The distal end of the VE cannula was attached to a micromanipulator clamped onto a ring stand. When visualizing the cannula under the dissecting microscope, the micromanipulator was used to insert the VE cannula into SC far enough to occlude the canal opening and prevent VE reflux. Gentle, steady pressure was applied to the VE cannula to introduce tinted VE into SC. Under the dissecting microscope, it was possible to track the progressive movement of tinted VE along the length of SC. Occasionally, progression of VE along the canal stopped but was restored by manipulating the cannula angle. After SC VE dilation, the quadrants were fixed in 4% paraformaldehyde for 16 h. Each quadrant was then returned to a Petri dish containing a base layer of paraffin and an overlying layer of a phosphate buffer solution.

Radial cuts were made in each quadrant to create approximately 500-µm thick segments. We initiated clockwise labeling of the segments as cuts were made to maintain relative orientation. We then prescreened the segments under the dissecting microscope and discarded those with a torn TM, damage to SC by the cannula, or an inadequately dilated SC.

An essential adjunct for screening involved placing the segments against a black background provided by an underlying black tape in the Petri dish. We then shined oblique light on the cornea. The illumination setup allows light to travel from the cornea through the limbal tissue, resulting in the SIV acting as a light pipe. The setup causes the otherwise semitransparent, diaphanous appearance to become readily visible against the background. The widely dilated canal and lighting arrangement permitted recognition of structural features and relationships distal to the TM, including the transparent SIV, CC, hinged flaps, septa, and the circumferential intrascleral channels. Segments deemed suitable at the dissecting microscope were further screened with a fluorescence microscope for the effectiveness of immunohistochemistry labeling. All adequately labeled segments with a dilated SC, radial orientation, and freedom from disruption during preparation were included.

### Immunohistochemistry

Segments were placed in a 24-well microplate and labeled with the following primary antibodies: CD31 (mouse antihuman CD31, endothelial cell; DAKO #M0823), Col Type 1 (rabbit anticollagen type 1 pAb; Calbiochem, #234167), and Col type 4 (rabbit antihuman collagen type 4; Chemicon #AB748) Table 1. Segments were also labeled with DAPI (stains dsDNA; Life Technologies, #D1306). The segments were first placed in PBS with sodium azide 0.02% and washed three times for 10 min on an oscillating platform. Next, segments were placed in 1% glycine for 1 h and then washed in PBS three times for 10 min. The segments were then placed in a blocking solution composed of 5% donkey serum secondary host, 1% BSA, and 0.3% Triton X-100 for 1 h. Two additional rinses were carried out with 1% BSA, 5% donkey serum, and PBS and then incubated in primary antibody for 48 h. After incubation, the segments were rinsed seven times for 5 min and then placed in secondary antibody for 2 h. Following secondary antibody incubation, segments were rinsed seven times for 5 min each and then placed in DAPI for 30 min. Finally, samples were rinsed three times for 5 min each. All primary and secondary antibody protocols were optimized with a dilution matrix, and secondary controls were performed.

**TABLE 1:** Antibody grid.

Primary antibody	Dilution	Secondary antibody	Dilution
CD31, mouse antihuman CD31, endothelial cell; DAKO #M0823	1:20	Alexa Fluor 647	1:200
Collagen type I (rabbit anticollagen type 1 pAb; calbiochem, #234167)	1:40	Alexa Fluor 555	1:200
Collagen type IV (rabbit antihuman collagen type IV; chemicon #AB748)	1:20	Alexa Fluor 555	1:200

### Imaging

After IHC was completed, but prior to clarification, each sample was loaded into our laboratory-designed chambers with multiple chamber heights. Height was chosen so that our free-floating samples did not move. Imaging was performed using a Zeiss LSM 510 META system using a ×20 dry objective and appropriate filter sets. The confocal microscopy fluorescence signal was optimized at the surface of each segment, an image was captured, and the initial confocal depth was recorded. Next, we scrolled through the segment to find the maximum confocal depth where an image of acceptable quality remained attainable. Acceptable quality was determined by the microscopist and defined as fluorescence easily distinguishable in the image with minimal noise in the background. The image was then captured, and the depth was recorded. After clarification, the segment was taken back to confocal imaging to repeat the protocol.

### Clarification

Determination of a practical clearing agent first involved dehydration and then immersion in one of the three agents: 1) glycerol with the immersion of segments in either of three concentrations, 50, 70, or 100%, using PBS as the diluent; 2) SCALE with three different protocols: a) segment immersion in Scale A2-4 Murea, 10% glycerol, and 0.1% Triton X-100, b) segment immersion in Scale-U2-4 Murea, 30% glycerol, and 0.1% Triton-X-100, and c) pulse/accelerated scale protocol, Scale A2 × 2 days, ScaleB4 (8 Murea, and 0.1%Triton-X-100) × 2 days, then back to Scale A2; and 3) immersion in a 1:2 ratio of benzyl alcohol–benzyl benzoate (BABB). After evaluation, BABB was chosen for further studies.

Once preclarification confocal imaging was complete, the segments were clarified using a two-step protocol consisting first of dehydration and second clarification. In the dehydration step, the samples were incubated in 30% ethanol for 15 min, moving progressively to 50, 70, 80, and 96%, each for 15 min. Finally, the segments were placed in 100% ethanol twice for 20 min.

After dehydration, segments were placed in a 1:2 ratio of BABB. The segments were then reimaged, first at the specimen surface and second at the maximum depth that provided a complete image—the reimaging permitted assessment of improvements in label detection post clarification. Segments had to be continually immersed in BABB for the entire experimental interval to remain optically clear. Standard ImageJ protocols optimized confocal image stacks and created 3D projections.

### Statistical Analysis

Descriptive statistics calculated the mean and standard deviations (mean ± SDs). Significant differences were analyzed using a paired two-tailed *t*-test. *p* < 0.05 was considered to be statistically significant. Excel v16.55 was used for analysis.

## Results

### Multiple Clarification Techniques—Effectiveness Comparisons

Segments were stored in Eppendorf tubes in respective solutions and maintained in a 4°C refrigerator. At 6 months, none of the specimens in the glycerol or SCALE had cleared. Specimens in BABB cleared within 1 day, and all the segments became observably clear enough for evaluation.

### Tissue Shrinkage Assessment With Benzyl Alcohol–Benzyl Benzoate Clarification

Clarification-dependent segment dimension changes were assessed by measuring 17 segments by light microscopy and ImageJ. The cornea segments were measured just anterior to the Schwalbe’s line. The mean segment width before clarification was 999 ± 352 µm [Range (R) 454–1,484 µm], and after clarification, it was 756 ± 234 µm (R- 338–1,177 µm). The average wedge width shrinkage at the corneal level was 242 ± 151 µm or 24.2% (*p* < 0.0001). The mean sclera segment width was measured at the junction of ciliary body attachment to the scleral spur. The mean scleral segment width before BABB was 733 ± 115 µm (R-506–948 µm), while after clarification, the mean scleral segment width was 685 ± 107 µm (R-518–921 µm). The change represents a decrease of 47.98 µm or 6.5% (*p* = 0.01). The mean SC area for 11 segments before clarification was 35,294 ± 20,923 μm^2^ (R-9,544–81,422 μm^2^). After clarification, the SC area was 29,423 ± 20,277 μm^2^ (R-7,462–79,247 μm^2^), representing a decrease in the mean SC area of 5,871 ± 6,185 μm^2^ or 16.6% (*p* = 0.01).

### Imaging Depth Assessment With Benzyl Alcohol–Benzyl Benzoate Clarification

**FIGURE 1:**
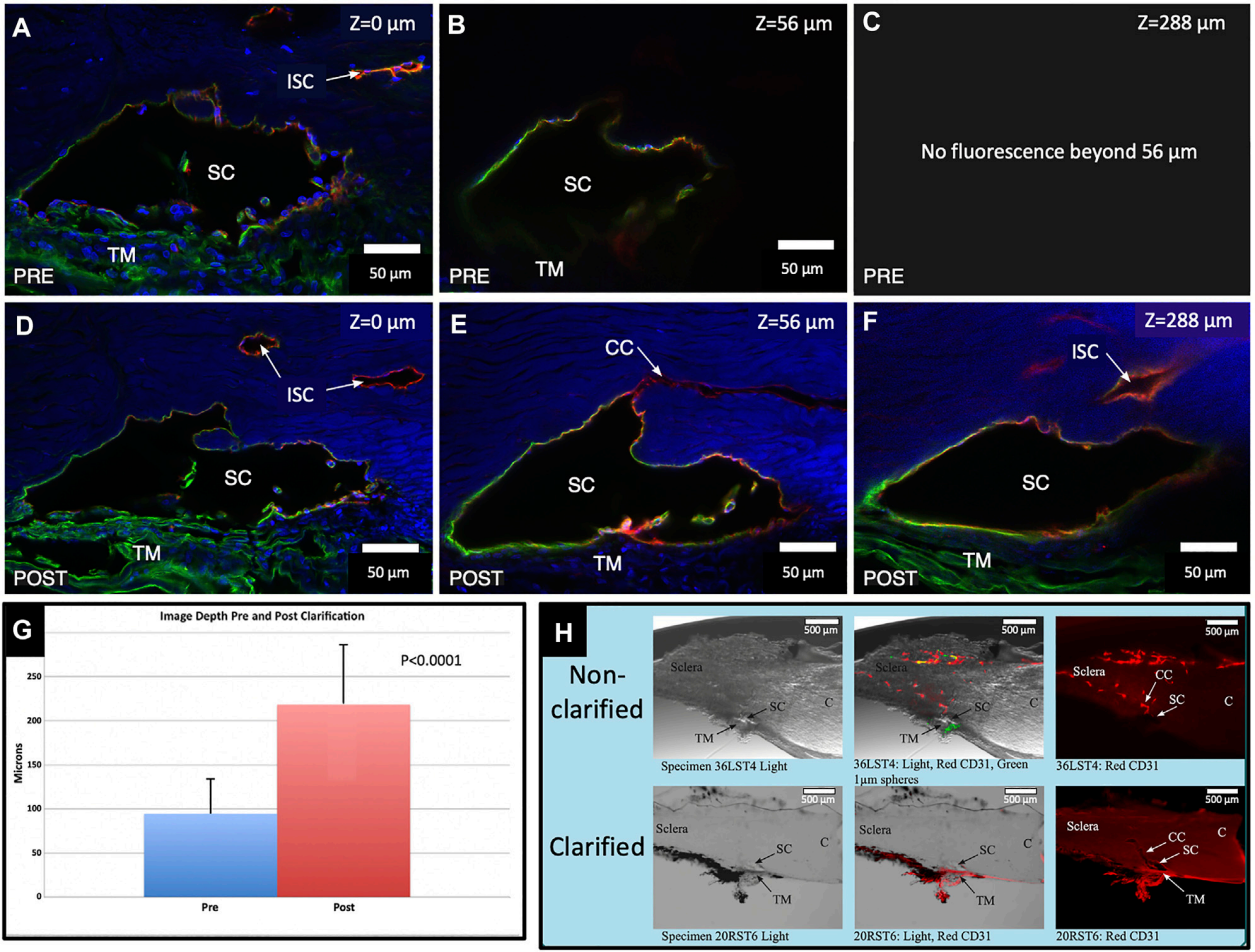
Clarification increases fluorescence imaging depth. Comparison of pre- and post-clarification of confocal microscopy z-stack image depth, following immunohistochemistry with merged channels of CD31 (red), Col type 4 (green), and DAPI (blue) **(A,D)**. The surface of the segment is labeled with a Z-axis of 0 µm. Subtle differences in composition noted in **(A,D)** are expected. We needed to position the segment in the well again after clarification. **(B)** Preclarification, the deepest practical imaging depth with an identifiable outline of SC was at 56 μm. **(C)** Fluorescence was absent beyond 56 µm. **(D)** After clarification, the fluorescent image brightness markedly improved. **(E)** Another image captured at B’s preclarification fluorescence depth limit is much brighter. Light passing along the lumen of SC permits retention of CD31 and the Col type 4 signals outlining the entire lumen of SC. **(F)** Deepest Z-axis fluorescence viewing depth post clarification was 288 µm into the stack. DAPI underwent marked signal attenuation as depth increased, whereas both the Col type 4 and CD31 signals appear undiminished. A Col type 4 signal is present within SC in images **(D,E)**. **(G)** Chart demonstrating the maximum imaged depth captured pre- and post-clarification. **(H)** Non-clarified and clarified segments compared with a light microscope channel (Left Panel), light and fluorescence microscope channels (center panel), and CD31 channel only (right panel). After clarification, the actual tissue becomes clear and transparent, which allows the fluorescent IHC label to be identified more clearly and deeply into the segment stack #38LSN10.

Preclarification, the average imaging depth attained in the total of 18 BABB clarified segments was 94.7 ± 38.9 µm (R-37.33–160.81 µm). Post clarification, the new average depth was 218.2 ± 67.5 µm (R-105.75–334.2 µm). The average increase in depth attained with clarification was 123.54 ± 82.95 µm or 176.9% (*p* < 0.0001), as illustrated in Figure 1.

### Schlemm’s Canal and the Distal Outflow Pathway Relationship Assessment After Benzyl Alcohol–Benzyl Benzoate Clarification

**FIGURE 2:**
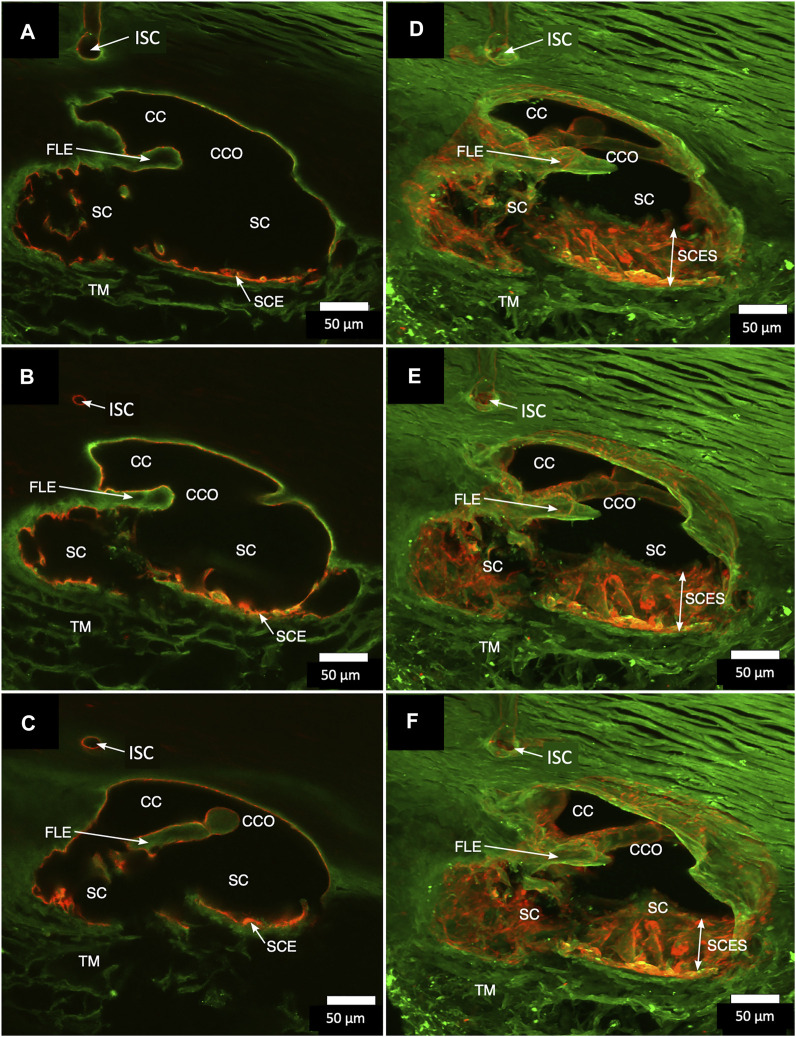
Projections display Schlemm’s canal flap-like collagenous extensions at collector channels. **(A–C)** are 2D while **(D–F)** are 3D projections of a 129-µm stack of merged CD31 (red) and Col type 1 (green) images. Viscodilation of Schlemm’s canal (SC), clarification, and deep stack projections characterize the organization and relationships of tissues surrounding collector channels (CCs) and their ostia (CCO). Flap-like collagenous extensions (FLE) from the sclera wall protrude into SC. The distal end of the FLE is not anchored, thus providing a hinged configuration. The FLE, CCO, and CC relationships are not easily conceptualized in the 2D images but become evident in 3D projections. TM is a trabecular meshwork #39RST13.

Confocal microscopy imaging depth increased after clarification, providing a valuable increase in tissue relationships while preserving the fluorescent IHC labels. The increased imaging depth permitted regional 3D projections of the TM, SC, and distal pathways. These 3D projections revealed complex relationships. SIV were cylindrical structures spanning SC that connected the SC inner wall endothelium and flap-like collagenous septa at CC entrances. Some SIVs instead attached directly to septa that divided SC from the CDSP. In Figure 2, CD31 and Col type 1 labeling demonstrate a collector channel ostium (CCO) and collector channel (CC) configuration at the external wall involving a flap-like collagenous extension (FLE). A circumferentially oriented intrascleral channel (ISC) was also parallel but separated from the canal by a thin septum.

**FIGURE 3:**
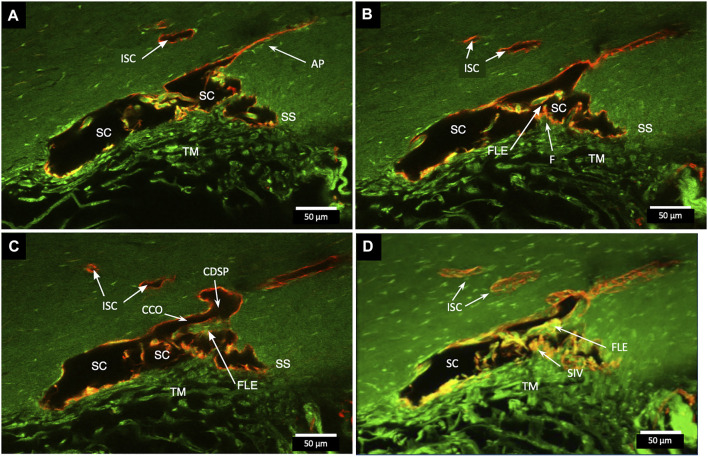
Relationships of the trabecular meshwork and flap-like extensions at collector channels. **(A–C)** Merged confocal CD31 (red) and native fluorescence (green) channel images demonstrate relationships of the trabecular meshwork (TM), Schlemm’s canal (SC), collector channels (CC), and flap-like extensions (FLE) at CC ostia (CCO). Serial images demonstrate a funnel region (F) that becomes cylindrical, forming a SC inner wall valve-like structure (SIV) that attaches to a FLE at the SC external wall. A CCO is visible and distal to the FLE, and a deep scleral plexus (CDSP) is seen. In **(A,B)**, an SC area leading to an intrascleral channel (ISC) has two individual endothelial cell layers in apposition (AP) consistent with a closed potential space. TM attachments to the scleral spur (SS) are visible **(B,C)**. **(D)** 3D projection of the 22 µm 2D region in **(A–C)** demonstrates the complex intrascleral vasculature of the SC and ISC connection #21L4-4.

In Figure 3, a CCO has one wall of the ostia demarcated by a flap-like extension (FLE) hinged to the sclera at one end and unattached at the other. The flap or leaflet is free to move because it is only connected at one end. The flap can respond to pressure-dependent TM movement because of the TM-SIV-hinged flap linkage. The merged confocal stack, imaged with native fluorescence and a CD31 label, was 192 µm thick. At 28 µm stack depth, a small CC entrance led to an appositionally closed CCO, recognizable as a double-lined endothelial structure (Figure 3A). At 62 µm stack depth, the CCO connection to SC widened, and the CC was no longer appositionally closed (Figure 3B). At 86 µm stack depth, the CC separated from SC but remained open (Figure 3C). The composite image of the stack revealed the funnel-shaped region of an SIV attaching to a flap-like extension at a CCO (Figure 3D).

**FIGURE 4:**
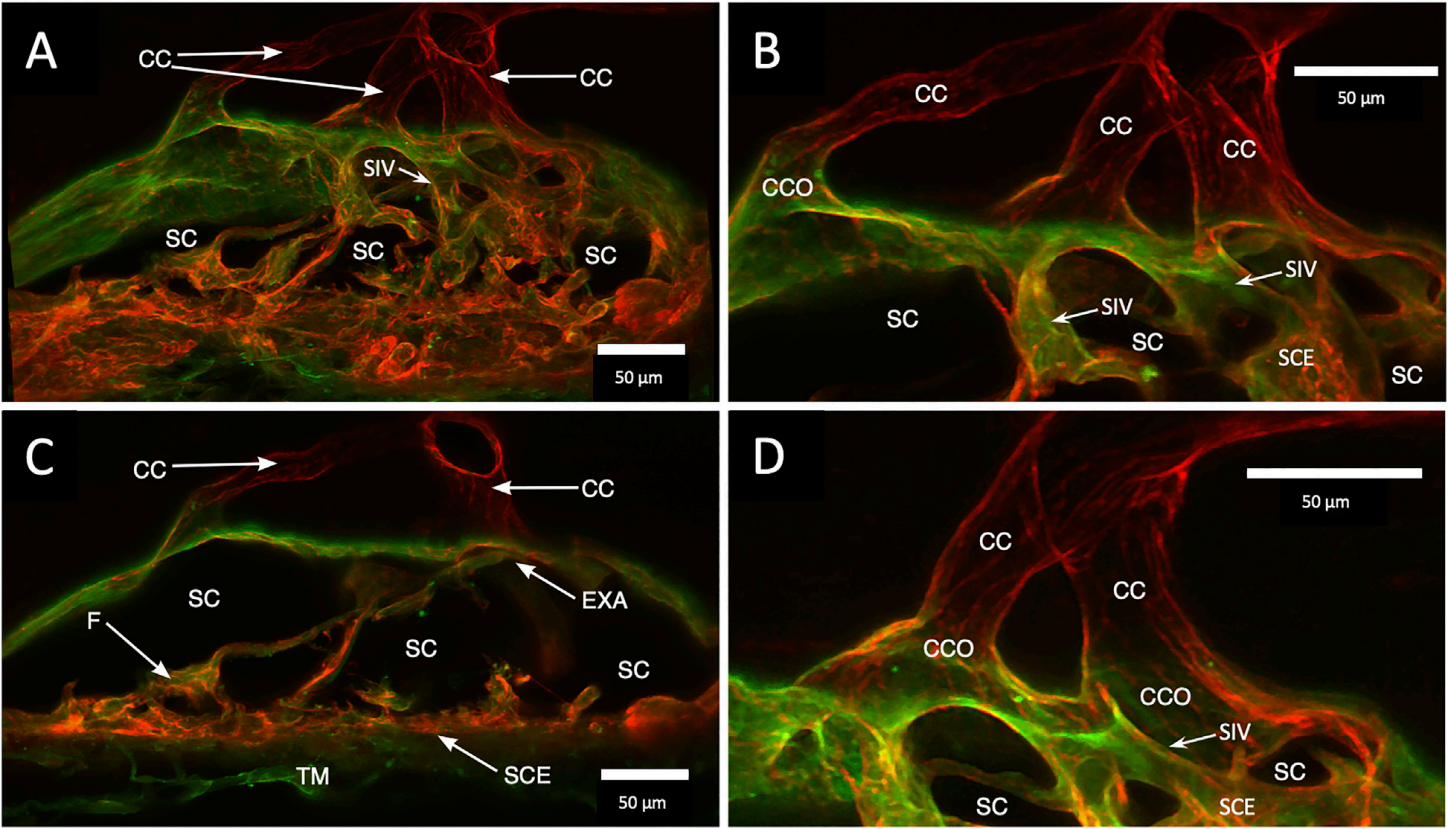
Clarification reveals Schlemm’s canal structure and collector channel relationships. **(A)** 112-µm thick confocal stack 3D projection of merged channels of CD31 (red) and Col type 1 (green). Clarification permits the appreciation of complex connections of the trabecular meshwork (TM) and Schlemm’s canal (SC) inner wall endothelium (SCE). SC inner wall valve-like structures (SIV) arise from SCE. The SIV attach to the SC external wall area (EXA) at collector channel ostia (CCO) that lead to collector channels (CC). **(B,C)** Subset stack of **(B)** 48 µm and **(C)** 18 µm. CCOs at the external wall lead to three CC traversing the sclera that join a circumferentially oriented intrascleral vessel. **(A–D)** SIV have a lumen apparent as voids in central sections of serial confocal image stacks **(D)** stack size is 42 µm. The SIV walls are labeled with CD 31 and Col type 1-like SC inner wall and form a funnel (F) developing into a cylinder attached to the SC external wall confirmed with serial confocal stack images #27L215.

**FIGURE 5:**
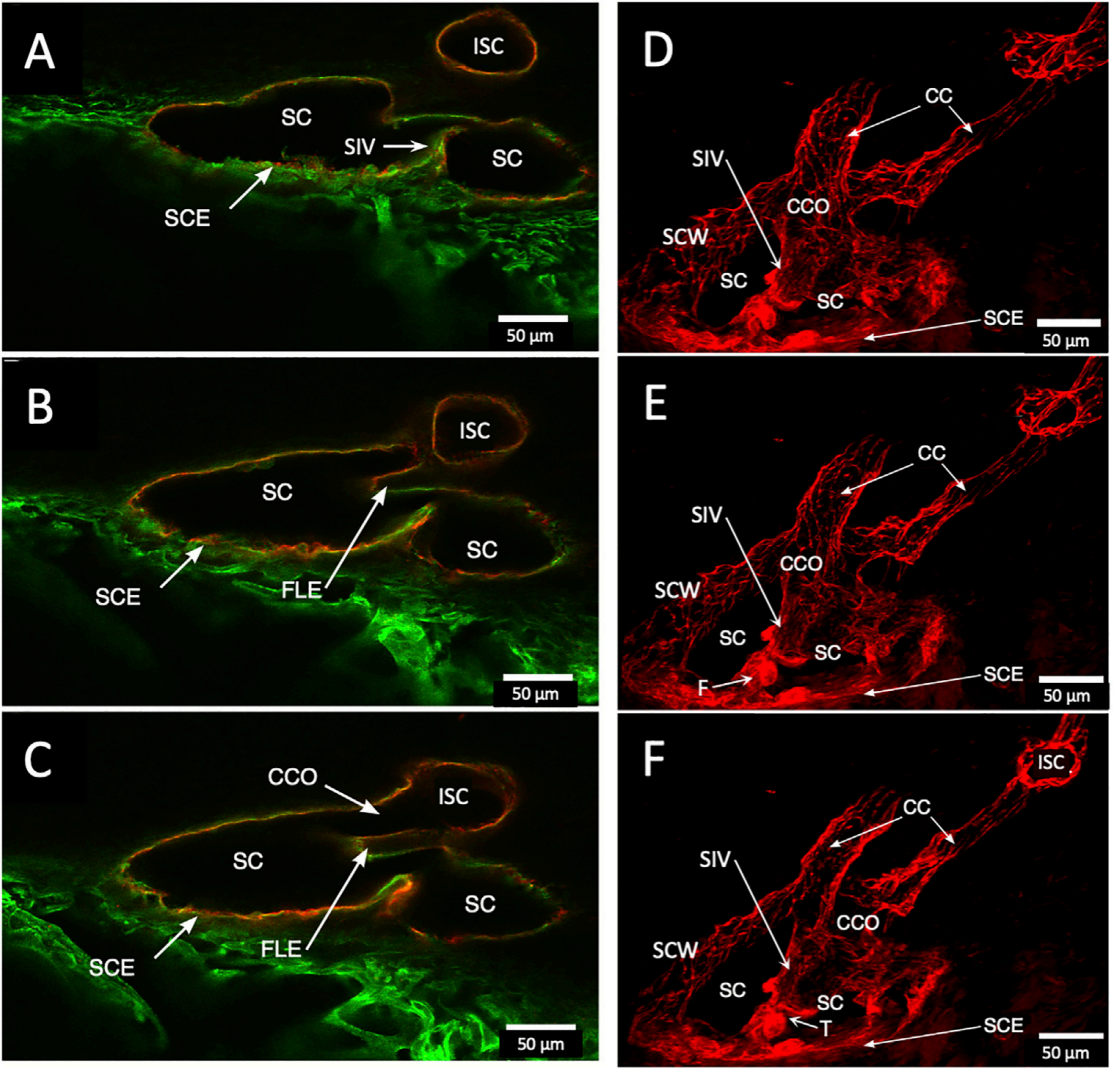
Schlemm’s canal inner and outer wall connections—conduits lined by the endothelium and basement membrane labels in two different specimens. **(A–C)** Merged CD31 (red) and Col type 4 (green) confocal images. Schlemm’s canal (SC), SC endothelium (SCE), collector channels (CCs), collector channel ostia (CCO), and relationship to flap-like extensions (FLE) are apparent. The funnel-shaped structure (F) develops into a cylindrical configuration to create a SC inner wall valve-like conduit (SIV) extending from SCE across SC to an FLE at a CCO. The FLE originates from the external wall sclera with a hinged arrangement forming one wall of a CCO. Notably, the cylindrical structure spanning SC approaches the external wall at the location where the CCO forms. **(D–F)** New specimen with confocal projection, following CD 31 labeling, outlines vascular conduits, including an SIV arising from SCE, to join collector channels passing to more distal intrascleral channels. **(F)** CC exits SC and travels distally to join an intrascleral channel (ISC), which runs circumferentially. T is a localized artifactual disruption of SIV continuity. **(A–C)** #37LSN715; **(D–F)** #20RIN8.

A confocal image projection in Figure 4 captures relationships of the entire outflow system using vascular endothelial adhesive (CD31) and collagen (Col type 4) labels. Structures captured include the SIV arising from the SC inner wall and three individual CC. The CC has lumen walls labeled with CD31, the vascular endothelial marker, and are continuous with the SC outer wall expressing the same endothelial label. Figure 4 projection also captures the convergence of three individual CC lumen to enter a single, larger, and more distal radially oriented vessel about 35 µm from the SC inner wall. Relationships between SC, CCO, and CDSP are also apparent in Figures 5A–C labeled with Col type 4 and CD31. Figures 5D–F are of the same CD31-labeled tissue. The images trace SIV from the SC inner wall to a CCO, CDSP, and more distal intrascleral channels, all luminal components of a contiguous aqueous pathway. The SIV lumen provides a continuous endothelial-lined pathway from the juxtacanalicular region to the intrascleral channels.

### Fluorescent Microsphere Perfusion and Schlemm’s Canal Inlet Valve-Like Structures Quantification

A preliminary study assessed the effect of the tracer size on the ability to enter the TM and SIV. Fluorescent microspheres with the diameters of 15, 10, and 8 µm were generally excluded from entry into the TM and were absent in the SIV. Microspheres of 2, 1, 0.5, and 0.2 µm diameters were found in the SIV. Active phagocytosis of 0.2 µm microspheres made interpretation of results difficult. We found the optimal sphere diameter for entry into the TM and SIV to be 1.0 and 0.5 µm. The two sizes had a similarly complete entry into the TM, so we grouped data from the two sizes for analysis.

The assessment of SIV distribution was limited to a subset of data in which all four quadrants of eyes were available, as summarized in the table as follows. There was a similar SIV frequency in the superior (1.27/segment) vs. the inferior (1.24/segment) hemispheres. However, the frequency of SIV-containing microspheres was 9% in the superior and 18% in the inferior hemispheres.

**FIGURE 6:**
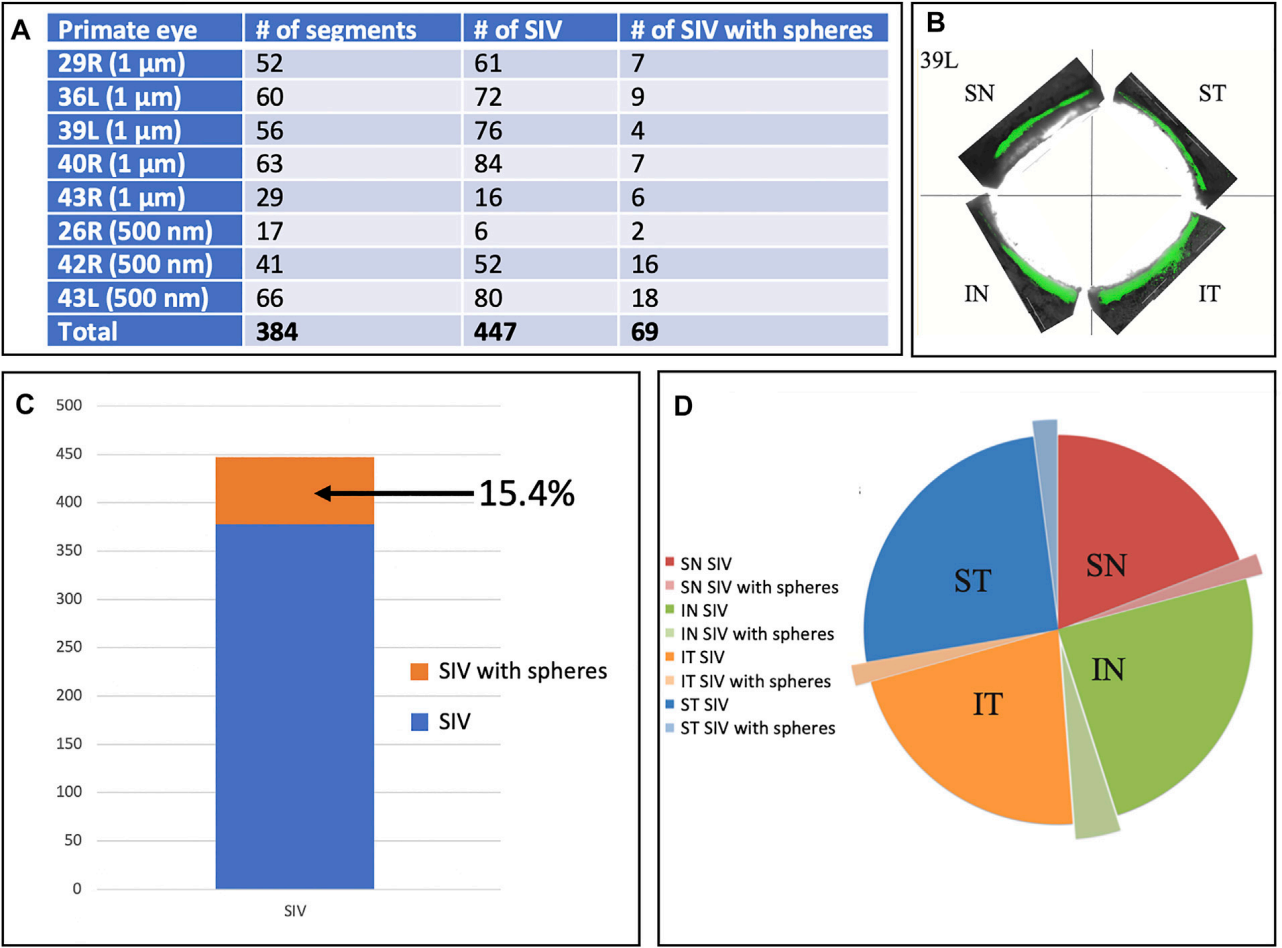
Schlemm’s canal inner wall valve-like structure frequency and microsphere presence. **(A)** Eight primate eyes were perfused with 1 and 500 nm fluorescent microspheres. The entire circumference of each eye was divided into 250–500 µm segments. The total number of Schlemm’s canal (SC) inner wall valve-like structures (SIV) in each segment and those containing microspheres were recorded. The mean number of the SIV/segment was 1.16 ± 0.38. **(B)** Light microscopy demonstrates the distribution of 1-µm fluorescent (green) microspheres in each quadrant in one eye. **(C)** Chart demonstrates the percentage of SIV with microspheres compared to the total documented SIV in the four eyes. **(D)** Pie chart shows the total SIV in each quadrant. The chart also shows those with microspheres in the four eyes where 360° of the SC circumference was examined. The microsphere distribution in SIV was similar in each quadrant.

In addition to analyzing SIV distribution per quadrant in an eye, as shown in Table 2, we also analyzed all eyes perfused with microspheres, including those without all four quadrants available. In this larger cohort, we analyzed 29 quadrants, in which there were 384 segments. In this cohort, we identified 447 SIV providing an average of 1.16 SIV per segment. A total of 447 SIVs were identified, and 69 or 15.4%, were found to have spheres within them (Figure 6). Eye 26 and 46R had one quadrant and two quadrants, respectively, available for the study because the other quadrants were used in unrelated experiments.

**TABLE 2:** Per quadrant analysis (only quadrants with all four segments examined were included in this analysis. Additional segments were imaged in the study but not included in the quadrant breakdown) SC inlet valve-like structures (SIV).

Quadrant	Segment	SIV	SIV w/microsphere
SN	74	92	10
IN	101	124	20
IT	96	120	24
ST	68	89	7
Total	339	425	61

Quadrant	SIV/segment	% of SIV w/microsphere
SN	1.24	10
IN	1.23	16
IT	1.25	20
ST	1.31	8
Average	1.25 ± 0.04	13.50 ± 5.50

**FIGURE 7:**
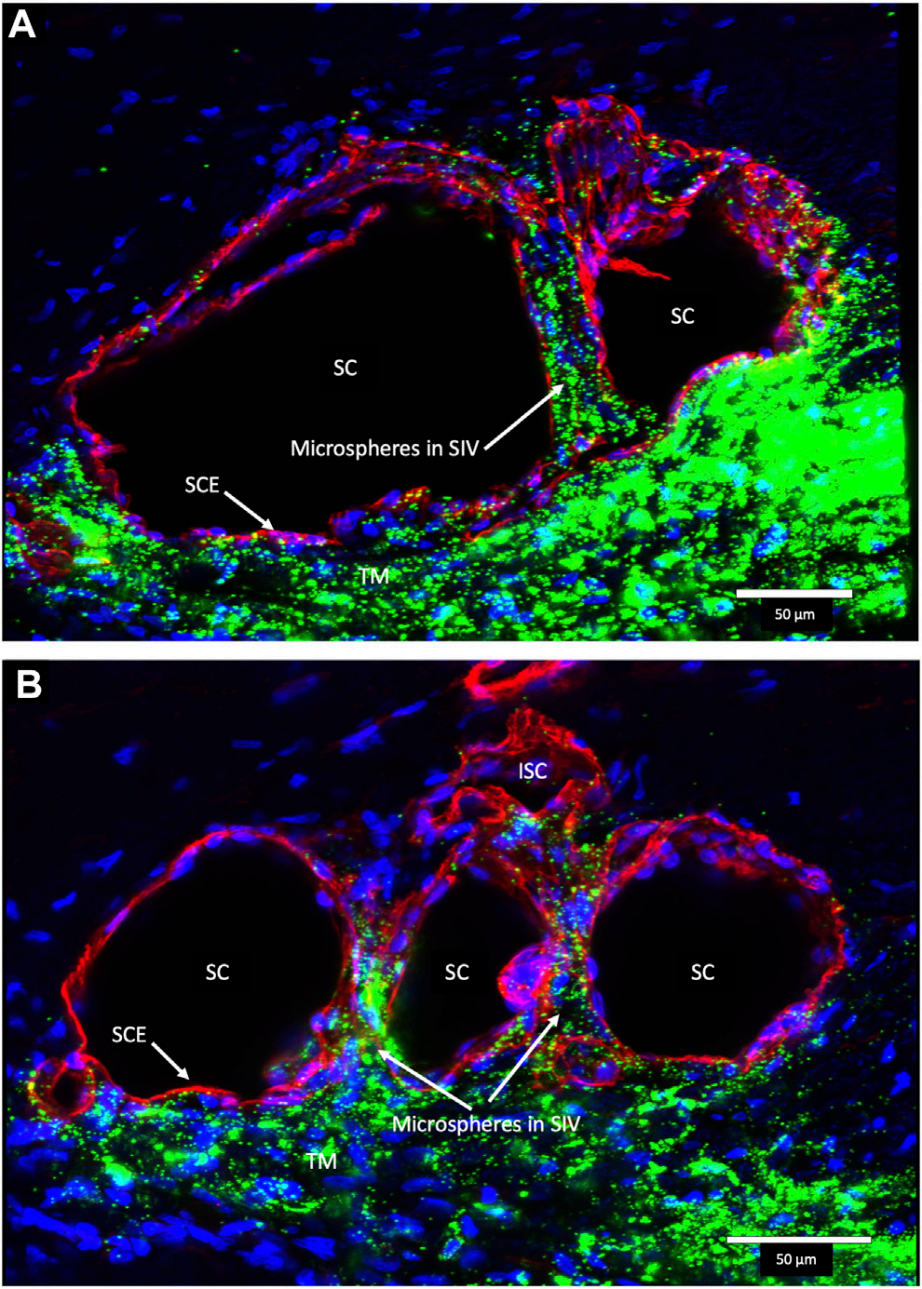
Fluorescent microspheres in Schlemm’s canal inner wall valve-like structures. **(A,B)** Merged CD31 (red), DAPI (blue), and 500-nm fluorescent microsphere (green) channels. **(A)** Stack size is 40 μm and **(B)** is 43 µm. Schlemm’s canal (SC) inner wall endothelium forms a valve-like structure (SIV). The SIV arise from the SC inner wall endothelium (SCE) that forms the outer wall of the trabecular meshwork (TM). The funnel-shaped configuration extends into SC to form a cylindrical conduit attaching to the SC external wall at collector channels (CCs). The TM is filled with fluorescent microspheres. The microspheres fill the juxtacanalicular region with the SIV and the SIV connections with intrascleral channels (ISC) Supplementary Video S1 **(A)** #43LST12; **(B)** #43LIT4.

**FIGURE 8:**
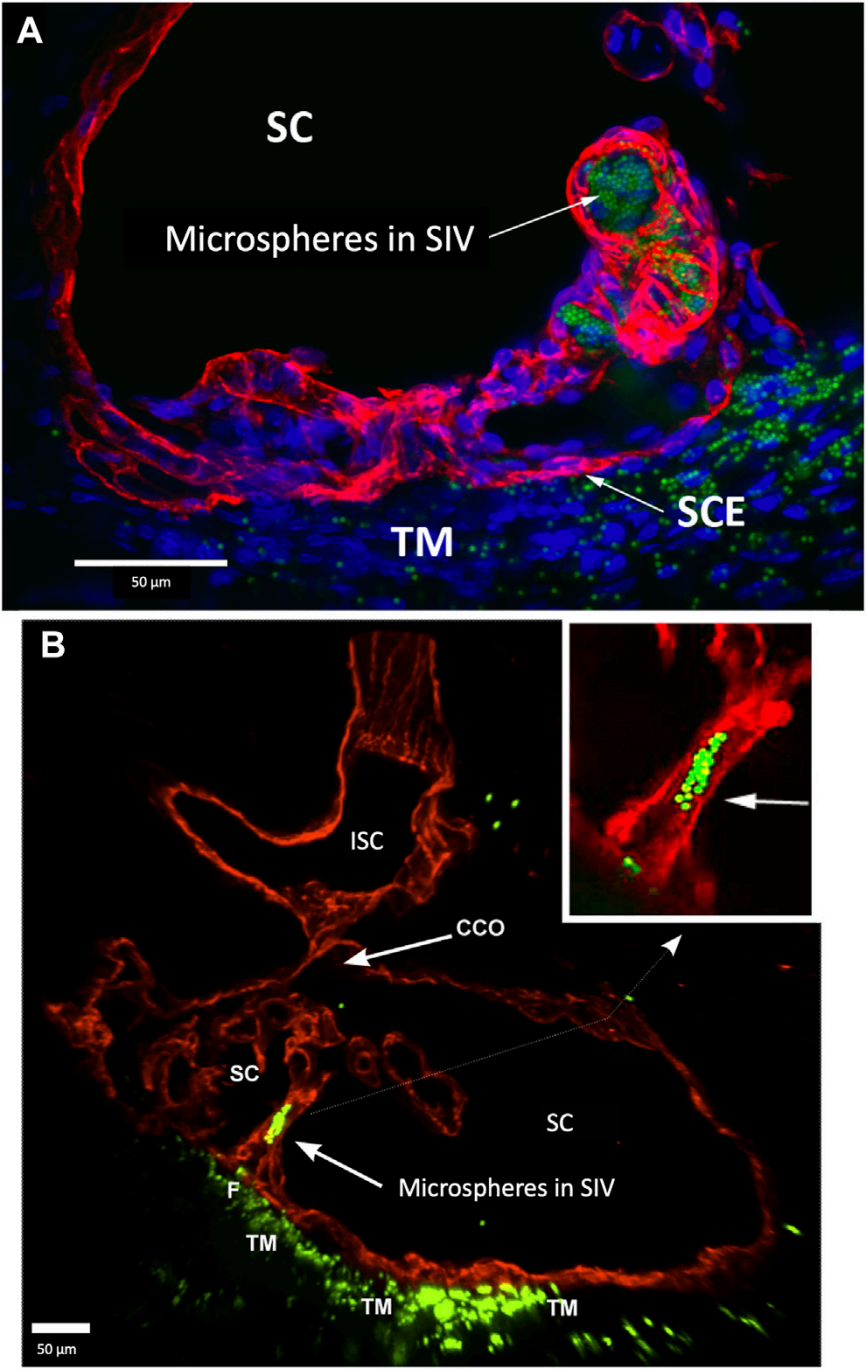
Perfused fluorescent microspheres enter the trabecular meshwork and a Schlemm’s canal inlet valve-like structure originating from Schlemm’s canal endothelium. **(A)** Merged CD31 (red), DAPI (blue), and 1-µm fluorescent microsphere (green) channels. The stack size is 60 µm. Schlemm’s canal (SC) inner wall endothelium forms a valve-like structure (SIV) oriented circumferentially in SC, permitting identification of a cross section through its diameter; fluorescent microspheres fill the SIV lumen. **(B)** Merged CD31 (red) and 1-µm fluorescent microsphere (green) channels. The stack size is 84 µm. The funnel-shaped configuration (F) at an SIV entrance extends into SC to form a cylindrical conduit attaching to structures arising from the SC external wall. The SIV contains microspheres, which were made more obvious in the inset image. A collector channel (CC) and its ostia (CCO) are apparent. Fluorescent microspheres are present in the trabecular meshwork (TM) in both **(A)** and **(B) (A)** #29RIN8; **(B)** #29RSN7.

**FIGURE 9:**
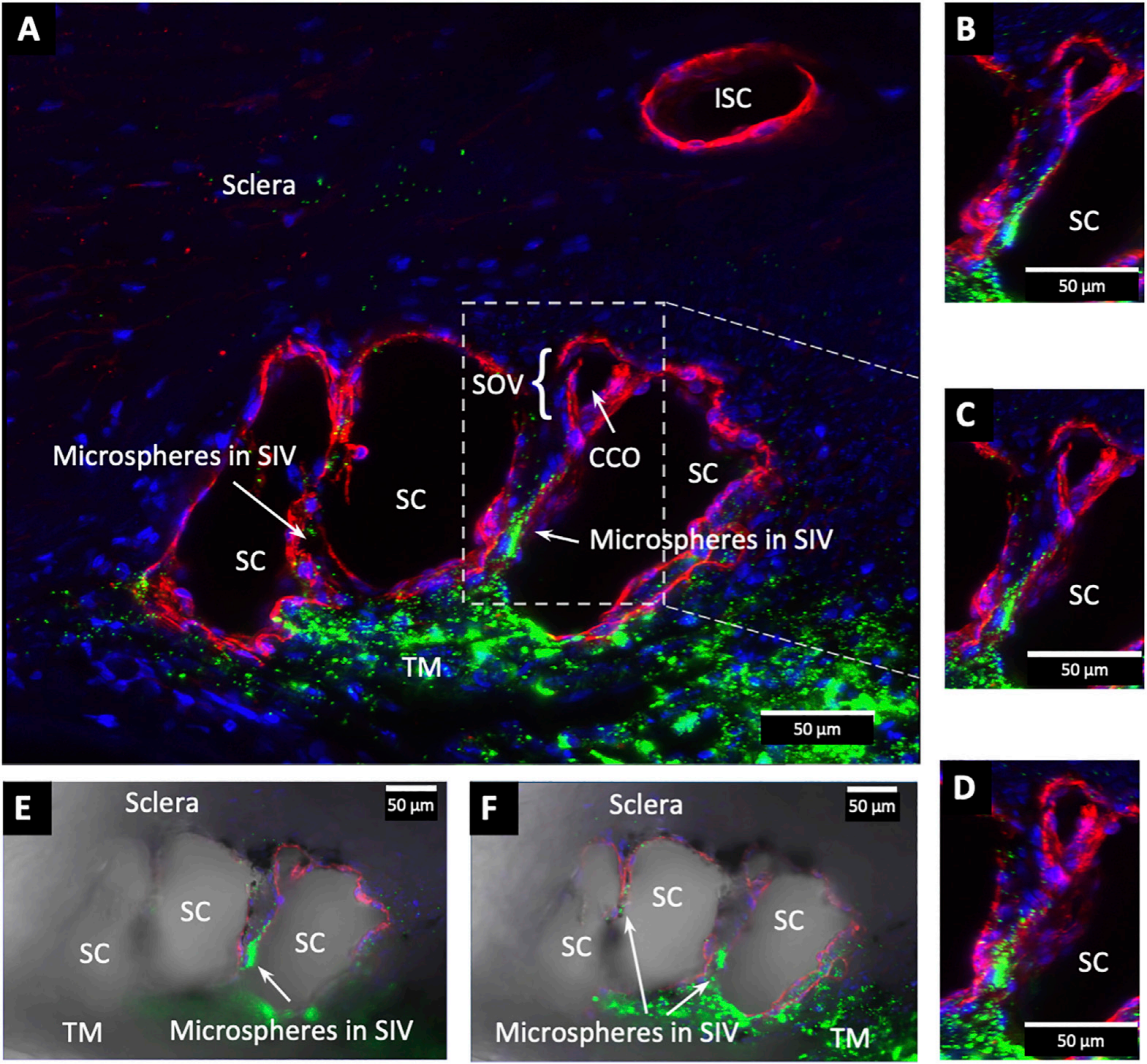
Fluorescent microspheres entering and exiting Schlemm’s canal inlet valve-like structures. **(A)** Merged CD31 (red), DAPI (blue), and 500 nm fluorescent microsphere (green) channels. Two Schlemm’s canal (SC) valve-like structures (SIV) attach to the external wall of SC. The complex distal attachment creates a valve-like structure at the SC outlet (SOV). **(B–D)** Same SOV structure rotated around the Y-axis. **(E–F)** Merged IHC channels add the Nomarski view to better show the structure of the SIV and their attachments to the distal wall of SC Supplementary Video S2 #43LIT12.

**FIGURE 10:**
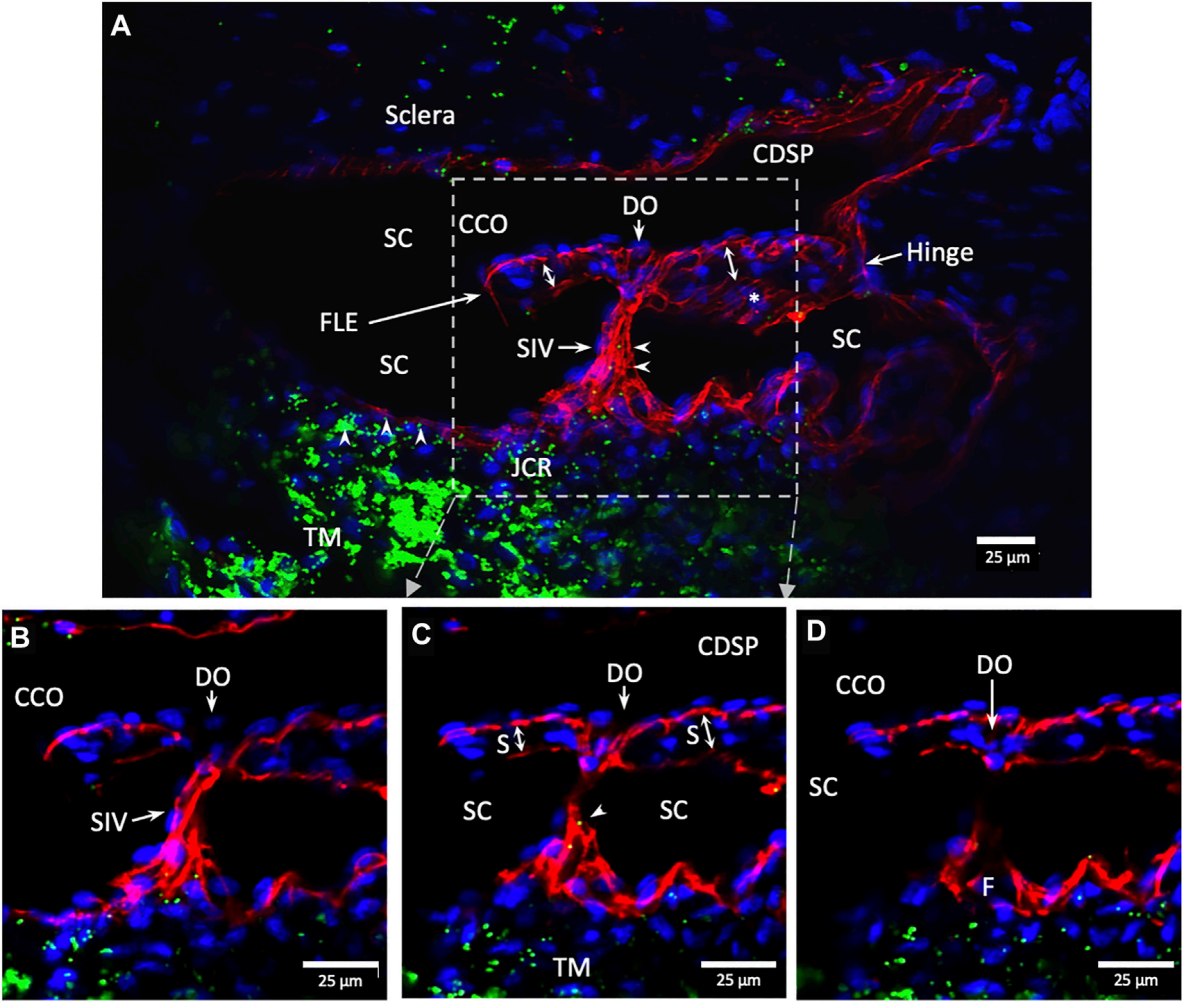
Conduit connects the juxtacanalicular region to collector channel ostia and its hinged flap. Merged CD31 (red), DAPI (blue), and 1-µm fluorescent microsphere (green) channels. **(A)** This 3D stack is 15 µm. A funnel-shaped region of the inner wall of Schlemm’s canal (SC) narrows to form an SC inlet valve-like structure (SIV). The SIV spans across SC and attaches to a septum (S) (double arrows). This septum has an unattached edge at a CC. In the cross section, it appears as a flap-like extension (FLE). The FLE is hinged at its scleral attachment. In this preparation, the open configuration of the collector channel ostia (CCO) provides communication between SC, CCO, SIV distal opening, and a circumferential deep scleral plexus (CDSP). The hinged flap can open or close, enabling it to function as a SC outlet valve. Green fluorescent microspheres (indented arrowheads) are at the SC internal wall, aggregate in the juxtacanalicular region, and are present in the SIV lumen. **(B–D)** represent the boxed area indicated in **(A)**. Serial non-stacked Z-axis images reveal that the SIV contains a continuous lumen extending from the juxtacanalicular region (JCR) across SC, passing directly through a septum where a distal opening (DO) enters the region of a (CCO). Asterisk indicates an outline of parallel endothelial cells on the undersurface of a septum. TM—trabecular meshwork and (F)—funnel-shaped region Supplementary Video S3 #40RIT13.

Confocal microscopy of the clarified segments containing microspheres helped characterize the complex SC cellular relationships of SIV and SOV. In Figure 7 **/** Supplementary Video S1 and Figure 8, the endothelium lining the walls of multiple SIV exhibit continuity with the SC inner wall endothelium. The SIV and SC inner walls also exhibit comparable CD31 vascular endothelial labeling. The SIV traverse SC where their CD31-labeled endothelial cell walls maintain continuity with cells of the external wall. Fluorescent microspheres lie directly adjacent to the SC inner wall endothelium in Figures 7–10. If SC’s inner wall was immobile, no microspheres would be expected to be found against the SC’s external wall. However, when SC inner wall endothelial cells distend in response to the pressure associated with the introduction of microsphere-containing fluid, the microspheres will necessarily enter the area of the cells newly undergoing deformation.

The SIVs are extensively filled with microspheres that can be traced from the juxtacanalicular region to the funnel-shaped SIV origins, then to their cylindrical portion, and finally to CC and distal intrascleral channels. Figure 9 **/** Supplementary Video S2 demonstrates the complex distal outflow relationships at the SC external wall where an SIV joins the region of a CCO, creating an SC outlet valve-like arrangement, the SOV. Figure 10 **/** Supplementary Video S3 demonstrates another image of an SIV attaching to the FLE, creating an SOV.

## Discussion

We reported a combination of techniques enabling the study of relationships of outflow system structures distal to the TM. These techniques included SC dilation with a viscoelastic, clarification of the tissues for enhanced depth of imaging, the use of the cellular adhesion molecule and basement membrane labels CD31, Col type 1, and Col type 4, and the introduction of fluorescent tracers that enter the distal outflow system through SIV. We found the combined techniques permit the use of IHC to explore cell–cell and cell–basement membrane properties of the SIV, SOV, their connections, and distal outflow system anatomic relationships, which is not amenable to study by traditional means.

### Technique-Dependent Requirements for Imaging of Schlemm’s Canal Inlet Valve-Like Structures and Schlemm’s Canal Outlet Valve-Like Structures

SC is not a pipe-like structure forming a straight-walled conduit. Instead, many endothelial-lined SIV projects from the SC inner wall and then spans between the TM and SC external wall, creating a highly complex geometry. In eyes fixed in hypotony or at positive pressure, the SC lumen is narrow (Grierson and Lee, 1975), and the SIV course circumferentially in the canal lumen (Johnstone, 1974; Johnstone, 2004; Johnstone et al., 2021). The circumferential orientation of the SIV results in requiring as many as 70 radially oriented 1- µm serial histologic sections to capture their entire length (Johnstone and Drance, 1984); laborious 3D reconstructions of serial images are then necessary to appreciate SIV structural properties and relationships (Johnstone, 2004).

The circumferential orientation of the SIV in SC and the configuration of attachments to the SOV are complex and not amenable to capture with 2D images of thin confocal sections. However, the dilation of SC causes the SIV to change from a circumferential to a more radial orientation. Even then, a montage of five of the lowest power electron micrographs is necessary to encompass the 2D dimensions of the TM, SIV, and SOV relationships (Johnstone, 2004). The aforementioned considerations make the characterization of the SIV impractical with traditional techniques.

Dilation of SC with viscoelastic prior to fixation was necessary to visualize and characterize SC, SIV, and SOV structure, composition, and relationship. Clarification with BABB permitted achieving greater imaging depth while retaining fluorescence properties. Combining SC dilation, clarification, and deep confocal imaging provided a global view of structural relationships not available with traditional approaches. The imaging depth permitted IHC and fluorescent microsphere labels to trace the path from the juxtacanalicular region through the SIV to the CC and intrascleral channels.

CD31 labeling indicated that the SIV walls are composed of an endothelial cell lining surrounding a lumen. Tracers demonstrate that the SIV lumen can function as a conduit for flow from the juxtacanalicular region to SC. The studies also demonstrated the structural continuity and comparable labeling properties of the SC inner wall endothelium, the SIV, their attachments to the SC external wall, the walls of the CDSP, and the more radially oriented intrascleral channels.

### Correlation of Study Results With Findings From Other Imaging Modalities

This study used IHC and tracers to identify SIV, SOV, and CDSP and ascribed functional significance to the entities. Such assertions require corroboration, which is available using evidence from multiple domains. Direct observation and manipulation in the operating room and at the dissecting microscope in human eyes, including published peer-reviewed video evidence, document the SIV anatomy, ability to distend rapidly, recoil, and alter CCO dimensions (Johnstone et al., 2021). The videos illustrated that investigators can confirm the SIV, septa, hinged flaps at SOV, and CDSP presence and behavior with human eyes and a dissecting microscope. Such direct observations require no assumptions and verify that the structures and their behavior are a physical reality of the natural world (Johnstone et al., 2021).

The SIV funnel shape arises from and is continuous with the SC inner wall endothelium and provides a continuous vascular endothelial-lined conduit to SC, as is documented with light microscopy (Shabo and Maxwell, 1972; Johnstone, 1974; Smit and Johnstone, 2002; Johnstone, 2004), SEM (Smit and Johnstone, 2002; Johnstone, 2004; Hariri et al., 2014; Johnstone et al., 2016; Carreon et al., 2017), and TEM (Johnstone, 2004). Red cell tracer studies demonstrated the presence of a lumen capable of carrying aqueous by light microscopy (Shabo and Maxwell, 1972; Johnstone, 1974; Johnstone, 2004) and TEM (Johnstone, 2004). Our current study using CD31 labeling further documents the composition and continuity of the SC inner wall endothelium, with the SIV walls and the presence of an SIV lumen.

An estimate of the number of SIV/mm was made using 218.2 µm, the mean sample depth of a radial section along the circumferential length of SC. The total SC length sampled in 384 segments was 83,788.8 µm, in which 447 SIVs were found, suggesting the presence of one SIV every 187.4 µm, or 5.33 SIV/mm. With this SIV frequency, a NH primate eye with an SC circumference of 33 mm (Li et al., 2012) would be estimated to have 175.89 SIV/eye. Assuming NH primates and humans have a similar SIV distribution, a human with a 38 mm SC circumference (Li et al., 2012) could be estimated to have 202.5 SIV/eye.

A prior study found 2 SIV/mm in primate eyes, consistent with ∼ 60 SIV in the entire circumference of the eye (Smit and Johnstone, 2002). Our study found 5.33 SIV/mm or about 175.89 in the entire circumference of an eye. There is a marked difference in the numbers, but imaging approaches differ. SIV can extend long distances circumferentially within SC. Our study may have been better able to detect the presence of SIV, but CFM microscopy depth limitations reduce the ability to ensure complete sampling of SC regions. In the prior study, we had a global view of millimeter long regions of SC compared to the 218 µm of our study. In the current study, we may have inadvertently counted the same SIV in two different segments, resulting in an overestimate of the SIV frequency.

Previous tracer studies in living primates involved red blood cells refluxed into SIV when EVP was raised above IOP. Pressure reversal resulted in primate red blood cells entering the SIV distal lumen and refluxing as far as the funnel region (Johnstone, 1974; Johnstone, 2004). Red blood cells introduced into the anterior chamber were found by light and transmission electron microscopy to fill SIV funnel entrances, passed through the cylindrical lumen, and reached as far as the SIV attachment to the SC external wall (Johnstone, 2004).

Our study identified fluorescent microspheres in 15% of the SIV. The microspheres in some segments filled the entire SIV funnel region, much of the cylindrical area, their contiguous CCO entrances, and more distal intrascleral channels. However, the lack of more frequent filling warrants an explanation, which is discussed under limitations.

**FIGURE 11:**
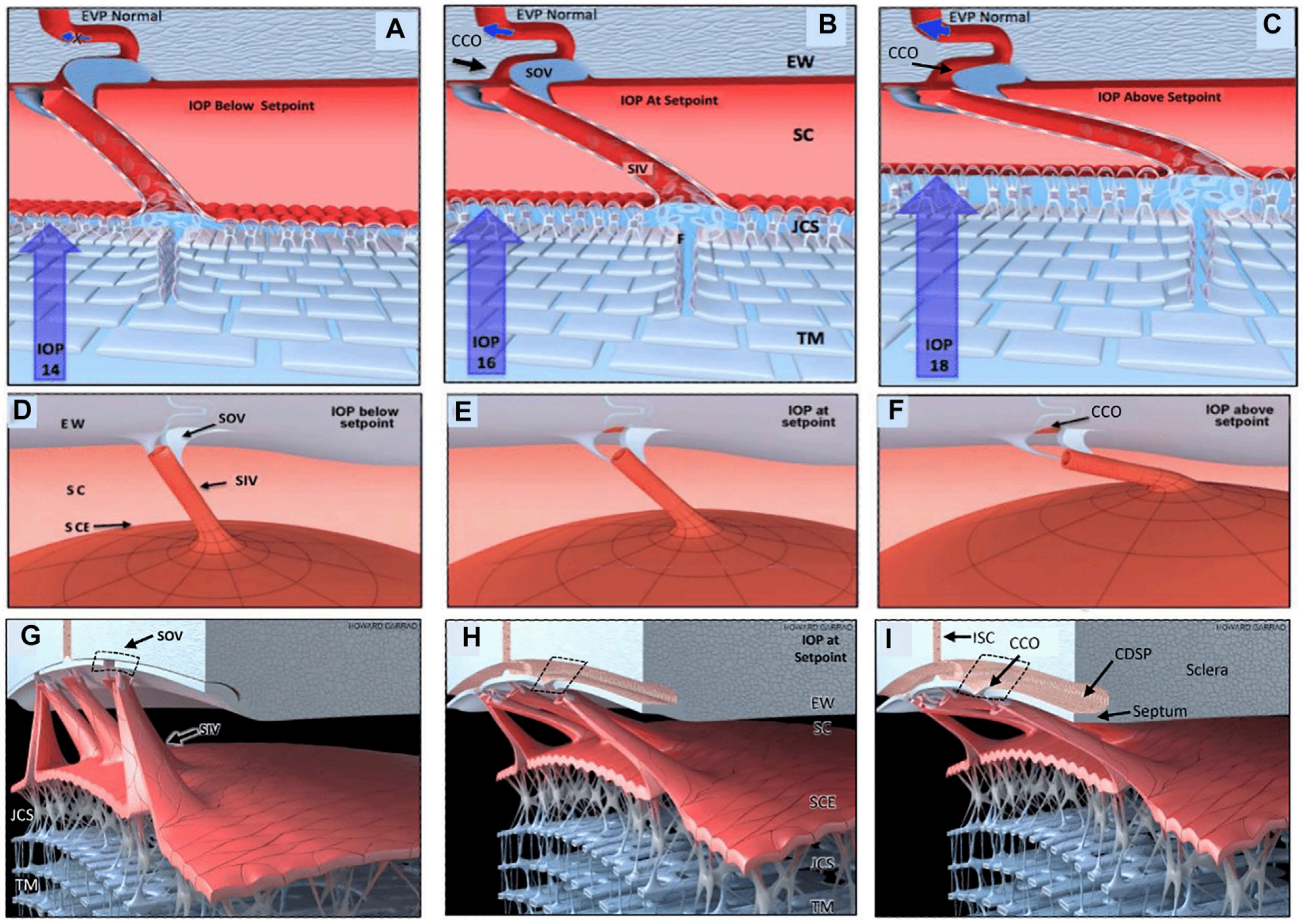
Depiction of the trabecular meshwork, Schlemm’s canal, and valve-like structure’s pressure-dependent relationships. Images **(A,D,G)** depict intraocular pressure (IOP) below a homeostatic setpoint, **(B,E,H)** at the setpoint, and **(C,F,I)** above the setpoint. In this provisional model, aqueous passes through the trabecular meshwork (TM) to the juxtacanalicular region (JCR) and then flows through Schlemm’s canal (SC) inlet valves (SIV) into the SC lumen at collector channel entrances or ostia (CCO) present at the SC external wall (EW). An SC outlet valve (SOV) consists of a mobile flap-like septum between SC and a circumferentially oriented deep intrascleral vascular plexus (CDSP). The SOV act as mobile flaps or leaflets at CCO. SIV connections to the TM provide a mechanism for pressure-dependent TM motion to control CCO dimensions. The SIV are oriented circumferentially in SC so that an intraocular pressure (IOP) increase that forces the TM into SC pulls the SOV open. The central column **(B,E,H)** depicts an IOP of 16 mm Hg as the optimal setpoint. An IOP of 14, below the setpoint, closes the CCO and SOV, while an IOP of 18 more widely opens them, providing a mechanical IOP-dependent control mechanism. The proposed model is based on verifiable evidence of SIV, SOV, and their motion in human eyes. However, the proposed regulatory mechanism is provisional, and its premises are subject to modification or rejection as new evidence emerges Supplementary Video S4.

Recent studies with high-resolution OCT (HR-OCT) have documented the presence of SIV. Funnel-shaped regions are seen that arise from SCE that then narrows to form a cylindrical or tubular conduit with a lumen to form the SIV. HR-OCT further demonstrates that the SIV cross the canal and connect to SC external wall-hinged flaps at CCO and septa (Hariri et al., 2014; Xin et al., 2016; Xin et al., 2021). HR-OCT shows that septa form hinged flaps at CC that, together with CDSP, form an outer wall valve-like arrangement (SOV) at CCO, as illustrated in Figure 11 **/** Supplementary Video S4.

The cylindrical SIV also undergo substantial elongation and shortening in milliseconds as SC dimensions change in response to changing pressure gradients (Hariri et al., 2014). The hinged flaps or leaflets attached to the SIV are mobile, move synchronously with the TM, and respond to pressure changes in milliseconds (Hariri et al., 2014). Mobility of the septa results in rapid pressure-dependent opening and closing of CDSP. Microvascular casts with clarification further demonstrated the CDSP adjacent to SC (Carreon et al., 2017; Johnstone et al., 2021). There may be two subsets of SIV, one that connects directly to hinged flaps at CCO and a second that attaches only to septa. The subset issue requires further study because some SIV have a lumen that passes directly through septa to the CDSP region. The arrangement results in the lack of appearance of a flap at CCO (Figure 10/Supplementary Video S4). The constellation of evidence from multiple sources corroborates and provides a framework for interpreting the current study’s findings.

### Adhesive Relationship Implications of Immunohistochemistry Findings

Rapid, substantial changes in axial dimensions of the SIV require the individual cells composing the cylindrically shaped walls to undergo substantial shortening and elongation in milliseconds in response to pressure gradient changes. The rapid large deformations in the SIV require robust cell–cell adhesive junctions. Otherwise, the cell walls of the SIV lumen would experience structural failure and separate. The SC inner wall endothelium and SIV are subjected to different tensional forces. Adhesion molecule distribution and the density can vary substantially within a vasculature. Potential variations in the adhesion molecule and cytoskeletal properties between the SCIW endothelium and SIV are relevant because SIVs link the TM and SOV. The currently described techniques allow exploring adhesion complexes in the structures distal to SC under changing pressure conditions.

The TM maintains connections to the SOV-hinged flaps through the cytoskeleton and junctional adhesion properties of the SIV. In studying resistance mechanisms, the SIV conduit configuration and their connections provide a means of controlling resistance at SOV. SIV connections make their tissue and cellular adhesive properties relevant in explaining normal outflow resistance. The functional behavior of the SC inner wall endothelium, cell walls of the SIV, and adhesive connections to the SOV are all dependent on optimized cellular adhesive mechanisms that may become abnormal in glaucoma (Webber et al., 2018).

Our current study supports the proposal that the balanced IOP-TM/ciliary body forces result in a unified proximal and distal outflow system maintained in a prestressed, tensionally integrated configuration by IOP. The concept posits that every cellular element experiences IOP-induced stress. The stress results in the strain that induces cell deformation. The linked IOP stress and resultant cellular strain convey instantaneous pressure information to the cell cytoplasm and nucleus. This massively parallel 3D information processing network can serve to inform cellular processes that use mechanotransduction mechanisms to maintain optimized cell adhesive properties (Johnstone et al., 2021).

### Schlemm’s Canal Outflow Resistance System

With the use of fluorescent tracers, we found the SIV and SOV to be coupled in a way that provides a continuous endothelial-lined pathway for aqueous flow. TM–SIV–SOV connections permit optimized TM elastance to maintain the septa and related SOV apparatus poised in a configuration that responds to mean pressures and oscillatory pressure gradient changes (Rohen et al., 1981; Johnstone et al., 2021).

The aqueous outflow system may have dual pump-conduit functions like the lymphatics. The linear motion of the TM can serve to induce pulsatile flow by changing SC pressure gradients. In addition, the linear pressure-dependent motion of the TM flow can control CDSP dimensions where Poiseuille’s law governs flow. The arrangement may permit the unified system to serve as a fluidic control system acting as an on–off switch, a fluid amplifier, or a fluidic logic gate with analogies in other circulatory systems. Of interest, the intrascleral CDSP loops have properties suggestive of the ability to act like Tesla valves responsive to oscillatory pulsations (Nguyen et al., 2021).

There are several ways the SIV, SOV/CDSP arrangement may function as a fluidic control system to regulate aqueous flow distal to the TM. This SC outflow resistance system, or SCORS, may provide an effective IOP regulatory mechanism, but additional work is necessary to clarify the details of control mechanisms. Resistance in the distal system at the level of the SIV and the CDSP may explain why EVP pressure levels are not achieved and why elevated IOP issues are not always resolved after MIGS surgeries that target the TM (Parikh et al., 2016).

### Limitations

Our tissue has acquired postmortem in primates of various ages and therefore likely health stages at the discretion of our primate center necropsy schedule. Because of this, we have no pre-existing eye examinations or intraocular pressure data. These confounding factors could have affected our study results. Before death, we had no role in animal care and did not administer any glaucoma-related drugs.

Our study has all the limitations of other *ex vivo* tracer studies with an absence of ciliary body tone, normal EVP, or ocular pulsations. Future studies can be carried out using *in vivo* fixation of NH primate eyes to circumvent the limitation. Our current study found 5.33 SIV/mm compared with 2.0 SIV/mm in a prior study (Smit and Johnstone, 2002). The prior study was specifically looking at tissue changes and results of viscocanalostomy. The techniques of the study may have destroyed structures within SC and thus may not be directly comparable to our current study. In addition, SIV can be oriented to span as far as 70 µm circumferentially within SC. Our technique did not provide the global view of the entire canal length afforded by the prior study (Smit and Johnstone, 2002). This study may have inadvertently double-counted some SIV present in more than one segment. Further studies are warranted to improve the frequency estimate.

Identification of only 15% of SIV with microspheres may also result from study limitations. EVP, ciliary body tone, and pulsatile flow are all likely to be determinates of normal SIV filling but are absent in our study. Moreover, we left the crystalline lens and ciliary muscle intact in our preparation. The internal portion of our organ culture apparatus pressed against the base of the ciliary body, while the external portion compressed the sclera. The ciliary muscle may shift forward, tending to move the scleral spur anteriorly and inward, resulting in closure or collapse of the canal. We set our perfusion at 24 mmHg, above normal physiologic pressure to try and encourage microsphere flow through the outflow system. In future studies, it would be valuable to assess microsphere passage using normal physiologic pressure. After perfusion, we dilate SC with viscoelastic, so our IHC would not provide information about the configuration at the time of perfusion. Future studies will explore how the organ culture system affects SC dimensions. Also, BABB was used to clarify the tissue, which we showed to cause some tissue shrinkage and may alter the dimensions and relationships of SC in our study.

After perfusion and prior to tissue fixation, we manually dilated the canal with viscoelastic, causing a reversal of transtrabecular pressure gradients, which is not typical of our habitual position. However, during physiologic activities such as gymnastics and yoga, SC fills with blood, and studies demonstrate that it undergoes configuration changes similar to what we achieved with viscoelastics (Weinreb et al., 1984; Friberg et al., 1987).

## Summary and Future Directions

As previously mentioned, we highlighted SC dilation, clarification, confocal microscopy, IHC, and fluorescent tracer techniques to visualize the TM, SC, SIV, CC, CCO, and CDSP in a minimally invasive manner that avoids separation of the inner and outer SC walls. The combination of techniques allows us to characterize the properties of individual structures, their connections, and their interdependence.

The juxtacanalicular region of the TM has long been portrayed as the location regulating IOP in normal subjects and the cause of dysregulation in glaucoma. Our study supports recent evidence that direct conduit-like pathways distal to the TM change dimensions in response to pressure are tensionally integrated, experience synchronous motion, and can provide a role in resistance regulation. Future studies applying these imaging techniques in human tissue are warranted. Future studies focusing on the distal outflow system tissue, cell–cell, and cell–ECM adhesive mechanisms may improve our fundamental understanding of resistance issues, enabling advances in targeted glaucoma treatment.

## Data Availability

The original contributions presented in the study are included in the article/Supplementary Material; further inquiries can be directed to the corresponding author.
